# Case Report: Somatic *GNAS* and *CACNA1D* mutations responsible for adrenal cortisol-producing adenoma with multiple aldosterone-producing micronodules—concurrence of primary aldosteronism and mild autonomous cortisol secretion

**DOI:** 10.3389/fmed.2026.1949358

**Published:** 2026-09-24

**Authors:** Lingna Deng, Yinchun Jin, Yihan Dong, Li Jiang, Zhongyu Wang, Haimin Xu, Jing Xie

**Affiliations:** 1 Department of Pathology, Ruijin Hospital, Shanghai Jiao Tong University School of Medicine, Shanghai, China; 2 Department of Pathology, The Affiliated Qingyuan Hospital (Qingyuan People’s Hospital), Guangzhou Medical University, Qingyuan, Guangdong, China

**Keywords:** adrenal cortical Cushing’s tumor, *CACNA1D*, *GNAS*, multiple aldosterone-producing micronodules, next-generation sequencing

## Abstract

**Background:**

The concurrent presence of primary aldosteronism (PA) and mild autonomous cortisol secretion (MACS) caused by adrenal lesions of a solitary cortisol-producing adenoma, accompanied by multiple aldosterone-producing micronodules (MAPM), is exceptionally rare. Moreover, there is a notable paucity of data regarding the molecular pathogenesis of this disease.

**Case presentation:**

We report a unique case of a somatic *GNAS*/*CACNA1D*-mutant cortisol-producing adenoma accompanied by MAPM and an aldosterone-producing nodule (APN) in the adjacent adrenal cortex. The patient was a 63-year-old woman with a 25-year history of hypertension and had a mass in the right adrenal gland. Detailed endocrine examinations revealed the concomitant occurrence of PA and MACS. Histologically, the main tumor was a well-circumscribed, encapsulated mass composed of neoplastic cells arranged in trabecular and nested patterns, exhibiting eosinophilic to vacuolated cytoplasm with uniformly round nuclei. Adjacent to the main tumor, a histologically similar 0.4 cm cortical nodule rich in clear cells was observed. Immunohistochemical analysis revealed diffuse CYP11B1 staining in the main tumor, whereas adjacent scattered cortical cell clusters and nodules exhibited distinctively diffuse CYP11B2 positivity. Next-generation sequencing results revealed missense mutations in the *GNAS* (p.R201H) and *CACNA1D* genes (p.F1131L).

**Conclusion:**

Our molecular findings from bulk tissue analysis identified somatic mutations in the *GNAS* and *CACNA1D* genes. Although the lack of microdissection precludes definitively assigning each variant to a specific pathological component, we hypothesize that these mutations may serve as key drivers for concurrent PA and MACS associated with cortisol-producing adenoma and adjacent MAPM/APN. Furthermore, our data support the emerging concept that primary aldosteronism can originate from MAPM rather than just from conventional adrenal cortical adenomas.

## 1 Introduction

Primary aldosteronism (PA) is the most prevalent endocrine cause of secondary hypertension, whereas mild autonomous cortisol secretion (MACS), although also a hypertensive disorder, occurs far less frequently (1, 2). The primary etiologies of PA include aldosterone-producing adenoma (APA) and idiopathic hyperaldosteronism (IHA) (3). Recent studies have further suggested that in patients with PA, IHA, or bilateral forms of primary aldosteronism might be typically caused by aldosterone-producing cell micronodules (APM) within the adrenal cortex (4–6). To ensure clarity and adhere to standardized nomenclature, we define these lesions based on their morphological characteristics: APM are defined as CYP11B2-positive lesions (<10 mm in diameter) composed of subcapsular zona glomerulosa cells that are morphologically indistinguishable from adjacent cortical cells on standard hematoxylin and eosin (H&E) staining; an aldosterone-producing nodule (APN) refers to a similarly sized (<10 mm) CYP11B2-positive lesion that is readily identifiable on H&E staining; and multiple aldosterone-producing micronodules (MAPM) denotes the presence of multiple subcapsular APMs accompanied by intermittent areas of normal zona glomerulosa. In patients whose secondary hypertension is attributed to an adrenocortical adenoma, concurrent biochemical evidence of PA and MACS may indicate co-secretion of aldosterone and cortisol from a single tumor, namely “Connshing syndrome” (7, 8). There are also case reports of two or more different adenomas on one or both sides independently secreting aldosterone and cortisol, leading to the simultaneous occurrence of PA and MACS (9–14). However, reports of a single cortisol-producing adenoma (CPA) accompanied by MAPM, resulting in PA combined with MACS, remain exceedingly rare.

At the molecular level, distinct types of adrenocortical lesions are characterized by specific driver-gene mutations. CPA most frequently harbors mutations in the *PRKACA* gene. APA is predominantly driven by mutations in the *KCNJ5* gene, whereas APM is most commonly associated with *CACNA1D* mutations (15). Notably, *GNAS* mutations are more frequently identified in adenomas related to “subclinical” mild autonomous cortisol excess (MACE), while these mutations are rarely found in adenomas causing overt Cushing's syndrome (16).

Here, we present a case of cortisol-producing adenoma with multiple aldosterone-producing cell clusters (APCCs) and a nodule in the adjacent cortex in a 63-year-old woman who has concurrent MACS and PA. Next Generation Sequencing (NGS) revealed missense mutations in the *GNAS* gene (exon8 c.602G>A, p.R201H) and *CACNA1D* gene (exon27 c.3393C>G, p.F1131L). We analyzed the similarities and differences in histopathology and molecular alterations between it and reported concurrent Cushing's syndrome/MACS and primary aldosteronism.

## 2 Case description

### 2.1 Clinical presentation

A 63-year-old woman was admitted to the hospital with a 25-year history of hypertension and a 3-month history of transient loss of consciousness. The syncopal episode lasted for 1 min and was not accompanied by seizures. Her medical history is notable for a cholecystectomy performed over 20 years ago and a hysterectomy conducted 18 years ago due to adenomyosis. Her elder sister had a history of hypertension but had never undergone an adrenal examination. The ambulatory blood pressure test showed that the 24-h average blood pressure was 150/93 mmHg, the daytime average blood pressure was 148/92 mmHg, and the nighttime average blood pressure was 156/101 mmHg. Two-dimensional computed tomography (CT) reconstruction revealed a right adrenal mass measuring approximately 2.0 cm (Figure 1).

**Figure 1:**
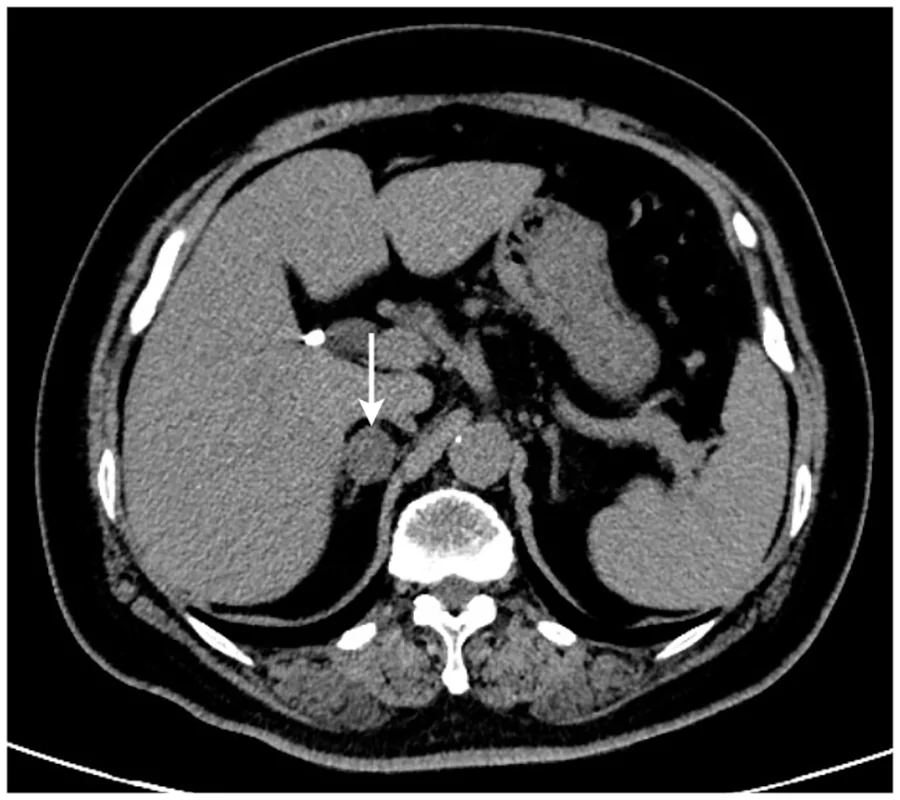
Two-dimensional CT image showing a 20 mm × 16 mm right adrenal tumor.

Serum potassium and sodium levels at admission were normal. As shown in Table 1, the patient exhibited an elevated plasma aldosterone level. In a simple standing test, her aldosterone-to-renin ratio (ARR) exceeded 38. The captopril challenge test showed a post-2-h plasma aldosterone level of 177.50 pg/mL. Moreover, bilateral adrenal venous sampling (AVS) was performed to assess the presence of lateralized aldosterone secretion (Supplementary Table S1). The absolute concentrations of aldosterone and cortisol were meticulously reviewed to avoid misinterpretation caused by autonomous cortisol secretion. Specifically, cortisol concentrations were significantly elevated in the right adrenal vein (RAV) at 302.60–307.16 μg/dL, compared to 18.25–27.66 μg/dL in the left adrenal vein (LAV) and 7.49 μg/dL in the inferior vena cava (IVC). The selectivity index (SI), defined as the cortisol ratio of the adrenal vein to the inferior vena cava, was 3.06 on the left and 40.7 on the right, both exceeding the threshold of 2, thus confirming successful selective catheterization.

**Table 1:** Laboratory data before operation, on the first day, and 1 and 2 months after the right adrenalectomy.

Peripheral blood	Before surgery	First postoperative day	One month after surgery	Two months after surgery	Reference range
Aldosterone (pg/mL)	484.71	129.40	289.98	602.87	29.06–332.78
Plasma renin	12.22	20.28	15.01	48.81	4–38
Simple standing test					
Supine aldosterone (pg/mL)	317.15	—	—	—	16.49–130.81
Standing aldosterone	340.86	—	—	—	29.06–332.78
Supine plasma renin (pg/mL)	2.50	—	—	—	4–24
Standing plasma renin	8.45	—	—	—	4–38
Aldosterone of CCT (pg/mL)					16.49–130.81
Baseline	333.85	—	—	—	
2 h	177.50	—	—	—	
24 h free UCOR (μg/24 h)	42.55	18.60	134.82	56.52	21–111
Plasma cortisol of 8 am (μg/dL)	8.89	1.89	3.20	5.27	6.70–22.60
ACTH (pg/mL)	12.30	11.80	—	—	7.0–65
Plasma cortisol of 1-mg ODST					6.70–22.60
After ODST (μg/dL)	6.62	—	—	—	
Serum potassium (mmol/L)	4.57	4.56	—	—	3.50–5.10
Serum sodium (mmol/L)	141	144	—	—	130–147
Bile acid (μmol/L)	11.6	—	—	—	1.0–10.0
BUN (mmol/L)	8.7	—	—	—	2.5–7.1
Creatinine (μmol/L)	144	—	—	—	53–97

ACTH, adrenocorticotropic hormone; BUN, blood urea nitrogen; CCT, captopril challenge test; ODST, overnight dexamethasone suppression test; UCOR, urinary cortisol.

Due to anatomical variations, the LAV drains into the left subphrenic vein, often resulting in the dilution of aldosterone and cortisol concentrations. In contrast, the RAV is shorter and empties directly into the inferior vena cava. To adjust for this discrepancy, the aldosterone-to-cortisol ratio (ACR) was calculated for each side. The absolute aldosterone levels were 1,678.52–2,753.44 pg/mL in the LAV and 1,205.88–1,773.26 pg/mL in the RAV. This yielded an ACR of 92.0–99.5 on the left side and 4.0–5.8 on the right side. The lateralization index (LI), derived by comparing the left and right ACRs, was 15.86–25.875 in this patient, significantly exceeding the diagnostic cutoff of 2, indicating left-sided dominant aldosterone secretion. With respect to cortisol, the concentration sampled from the right adrenal vein was substantially higher, exceeding the left-sided concentration by a factor of 10.94–16.83. This significant gradient suggests a potential right-sided dominance in cortisol production. Although the patient's 24-h free urine cortisol and plasma cortisol levels were within the normal range, plasma cortisol remained unsuppressed after an overnight 1-mg dexamethasone suppression test (ODST). Based on these results, the patient was diagnosed with MACS combined with primary aldosteronism.

The clinical rationale for performing laparoscopic resection of the right adrenal gland was based on the structural and functional divergence of the lesions. The right adrenal gland harbored the 2.0 cm macroscopic tumor identified on CT and was confirmed as the definitive source of the autonomous cortisol excess. Resection of this dominant right-sided mass was suggested to resolve the MACS, with a multidisciplinary consensus that the unmasked left-sided aldosterone hypersecretion would be actively monitored and managed medically after the operation. The clinical evolution and postoperative follow-up showed significant improvement, with no postoperative complications. As shown in Table 1, the plasma cortisol and 24 h free urinary cortisol levels significantly decreased after the surgery. Aldosterone levels decreased on the first day and 1 month after the operation, but significantly increased 2 months after the operation, accurately reflecting the persistent bilateral/left-dominant aldosterone production suspected from the absolute AVS values. At the 11-month clinical follow-up, the patient reported favorable clinical outcomes, with her blood pressure well-controlled at approximately 110–120/70–80 mmHg. Her antihypertensive medication requirements were adjusted to a daily morning regimen of oral nifedipine sustained-release tablet (20 mg) and amlodipine sustained-release tablet (5 mg). A comprehensive timeline summarizing the patient's major clinical events, diagnostic work-up, and follow-up is shown in Supplementary Figure S1.

### 2.2 Pathological findings

Macroscopic examination revealed a well-demarcated mass (2.5 cm × 2.0 cm × 1.8 cm) in the right adrenal gland of the patient. The cut surface was solid, of medium consistency, and exhibited a variegated grayish-yellow or grayish-brown appearance. At low-power magnification, the tumor appeared well-circumscribed and encapsulated, with compressed and mildly hyperplastic adrenal tissue visible externally. Internally, neoplastic cells were arranged in trabecular and nested architectures (Figure 2A). Under high-power examination, these cells exhibited a mixed population with eosinophilic and vacuolated cytoplasm. The nuclei were uniformly round and exhibited rare mitotic activity (Figure 2B). Interestingly, a nodule of 0.4 cm was observed within the adrenal cortex adjacent to the tumor. Its histological features closely resembled those of the dominant mass, but it contained more clear cells (Figures 2C,D).

**Figure 2:**
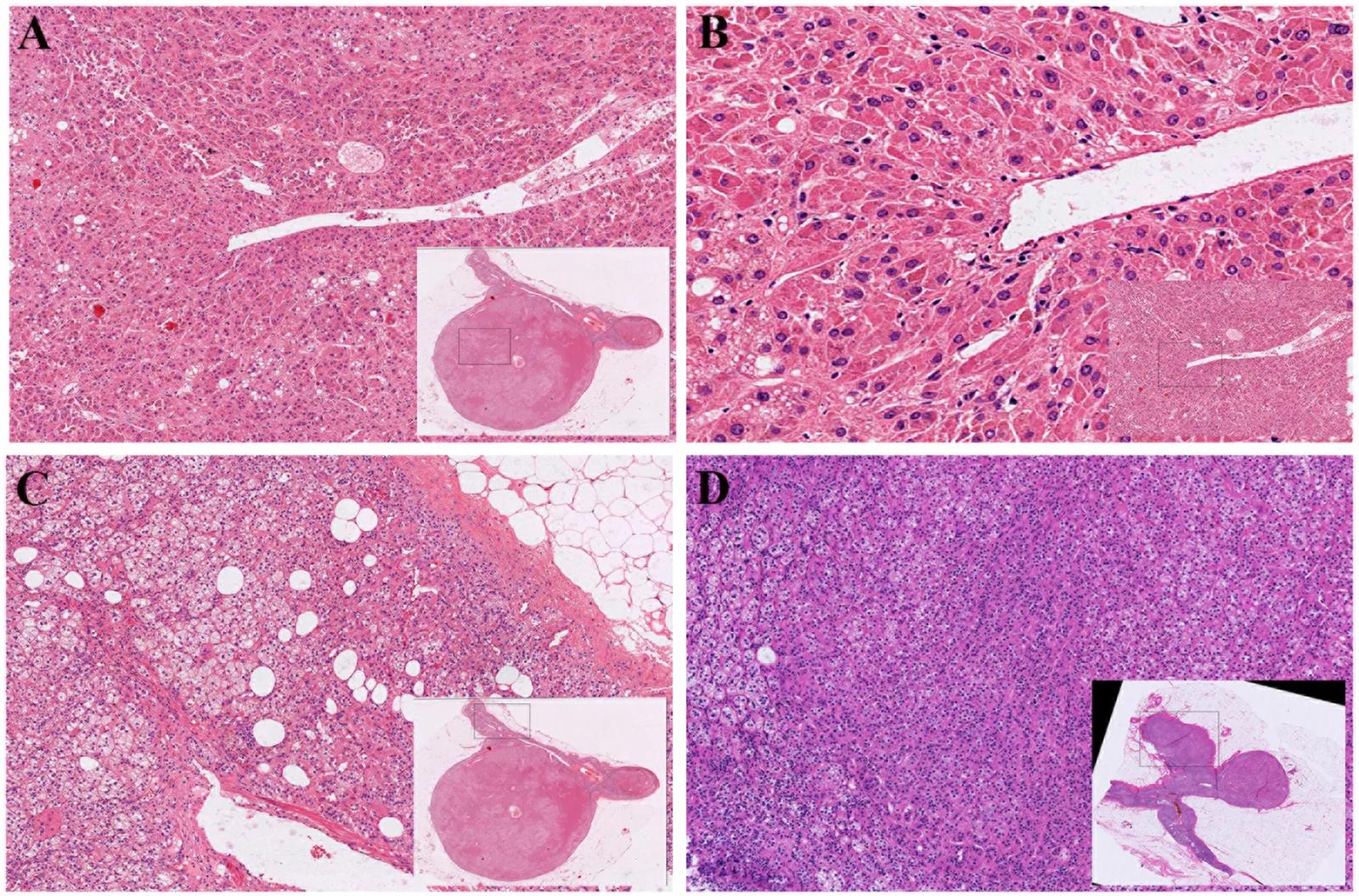
CPA accompanied by MAPM. **(A)** Neoplastic cells of CPA are arranged in trabecular and nested architectures under low-power magnification. **(B)** Under high-power examination, these cells exhibited a mixed population with eosinophilic and vacuolated cytoplasm. The nuclei were uniformly round and exhibited rare mitotic activity. **(C)** The adrenal cortex adjacent to the CPA appears histologically unremarkable. **(D)** A 0.4 cm adrenal cortical nodule located adjacent to the CPA.

### 2.3 Immunohistochemical findings

To determine the functional nature of the resected tumor, we performed immunohistochemical (IHC) staining on the tumor cells (Supplementary Table S2). The tumor cells demonstrated variable expression of CD56 and Inhibin and were negative for MelanA and CyclinD1. These immunoprofile findings support an adrenocortical origin. The MIB1 (Ki67) labeling index was 2%, and the P53 expression was heterogeneous. Within the main tumor, diffuse positivity for CYP11B1 and steroidogenic factor-1 (SF-1) was observed, while CYP11B2 was only focally and weakly expressed (Figures 3A,B). In contrast, multiple scattered cortical cell clusters (Figure 3C) and a 0.4 cm nodule (Figure 3D) surrounding the tumor exhibited unique focal localization positive for CYP11B2 expression. The immunohistochemical findings suggested that the main nodule was a Cushing's tumor. The remaining adrenal cortical tissue contained MAPM/APN.

**Figure 3:**
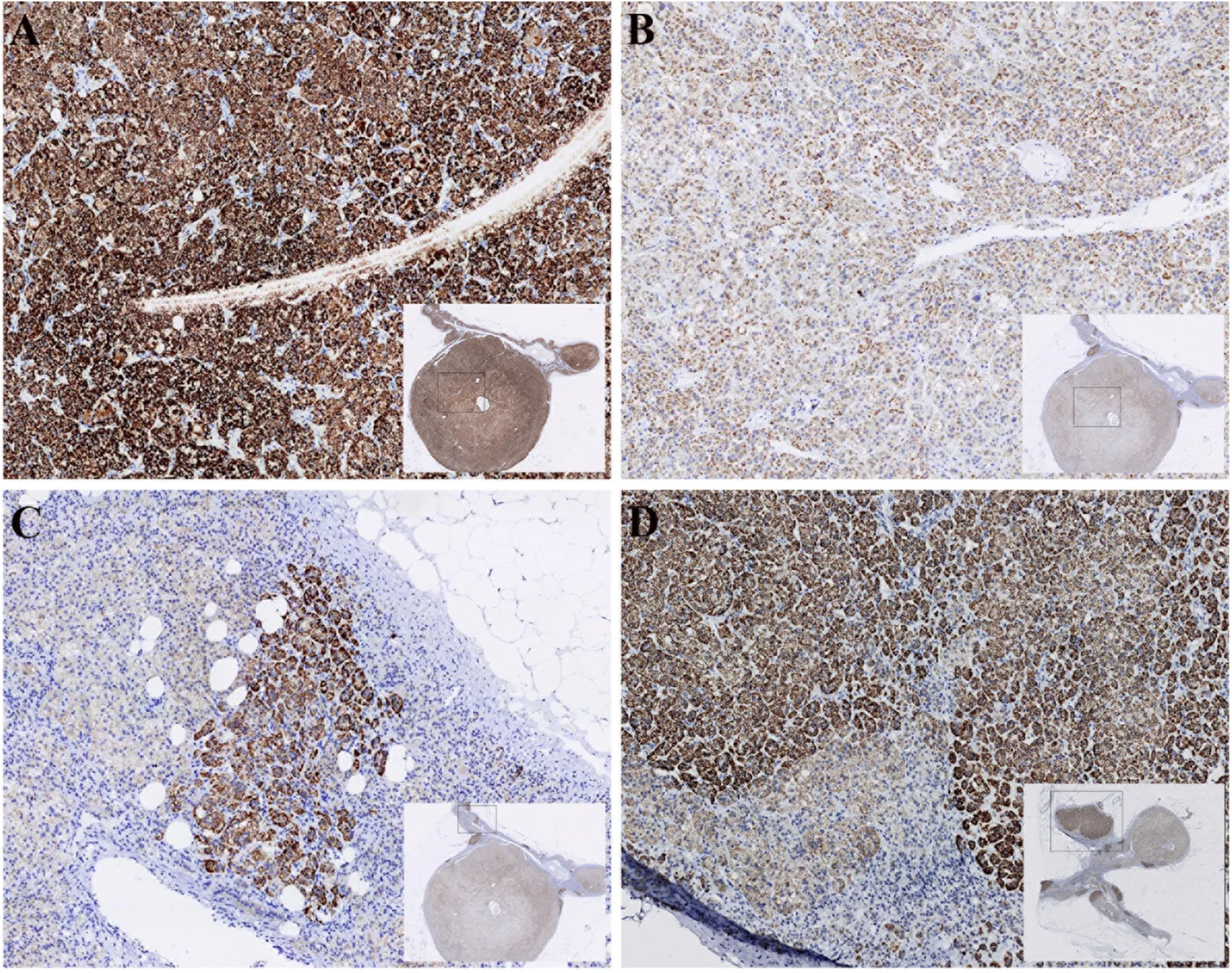
Immunohistochemical biomarkers in CPA accompanied by MAPMs and APNs. **(A)** Diffuse positivity for CYP11B1 in CPA. **(B)** Focal and weak expression of CYP11B2 within the CPA. **(C)** Unique focal localization positive for CYP11B2 in multiple scattered cortical cell clusters surrounding CPA. **(D)** A 0.4 cm adjacent adrenal cortical nodule also demonstrated diffuse CYP11B2 immunoreactivity.

### 2.4 Molecular findings

To determine whether the patient had genetic alterations, we conducted combined genetic testing on DNA extracted from the patient's right adrenal tumor (Supplementary Table S3). DNA was extracted using the QIAamp DNA FFPE Tissue Kit (Qiagen, Germantown, MD, USA). All procedures were performed according to the manufacturer's instructions. DNA samples were analyzed using the S5 platform for combined genetic testing (Thermo Fisher, Waltham, MA, USA).

Analysis of the right adrenal gland identified two somatic missense variants: *GNAS* (exon8 c.602G > A, p.R201H) and *CACNA1D* (exon27 c.3393C > G, p.F1131L). The frequencies of the variant alleles (VAF) were 30.8% and 51.0%, respectively (Supplementary Figure S2).

## 3 Discussion

The patient in our case presented with a history of hypertension. Endocrinological tests indicated hyporeninemic hyperaldosteronism, and CT revealed a solitary right adrenal mass. Based on clinical, biochemical, and imaging data, the mass was initially considered to be an aldosterone-producing cortical adenoma. However, the AVS did not fully support this diagnosis. A subsequent dexamethasone suppression test revealed elevated cortisol levels. Histopathological examination revealed a single CPA in the right adrenal gland with a 0.4 cm nodule around it. Immunohistochemical analysis revealed that CYP11B1 and CYP11B2 were expressed in CPA and MAPM/APN cells, respectively. Therefore, the final diagnosis was CPA combined with MAPM/APN. This finding was consistent with the results of AVS, suggesting that the right side is the dominant side of cortisol. Further molecular testing revealed that this case had both a *GNAS* somatic mutation of the driver gene commonly found in CPA and a *CACNA1D* somatic mutation, a potential pathogenic variant frequently seen in MAPM/APN. Because sequencing was performed on bulk tissue samples from single sites, we could not definitively localize the precise cellular origin of each variant. However, these genetic results, combined with the distinct CYP11B1 and CYP11B2 immunohistochemical staining patterns, support the possibility that the patient's hypertension was not caused by the co-secretion of aldosterone and cortisol from a single tumor. Instead, they favor the hypothesis of two independent hormone-secreting lesions. This discovery provides new ideas for the precise diagnosis and treatment of secondary hypertension. Furthermore, although AVS is of significant value in the diagnosis of PA, clinicians should still actively screen for hypercortisolemia to facilitate accurate interpretation of AVS results.

Atypical clinical manifestations and laboratory findings in combined PA and MACS pose diagnostic challenges (17). When a patient presents with features of both PA and MACS, it is necessary to determine whether a single adenoma is co-secreting multiple hormones or whether two distinct lesions are secreting hormones independently. Although several case reports have described unilateral adrenal lesions associated with concurrent elevations of both hormones (18–20), the majority relied on indirect evidence, such as immunohistochemical co-expression of CYP11B2 and CYP11B1 within the same lesion. However, such findings do not exclude the possibility of microscopic or radiologically occult multiple nodules, each responsible for one hormonal axis. Moreover, the absence of concurrent driver mutations in both steroidogenic pathways within a single lesion weakens the argument for true monoclonal co-secretion. Although a co-secreting adenoma was proposed, the case reported by Hu et al. showed only CYP11B2 positivity and was negative for CYP11B1. The authors rationalized this by suggesting that although the production per cell was low, the overall amount was still large (20). Similarly, other reports lack clonality studies or mutation profiling to definitively establish a single tumor origin for both hormones.

The coexistence of hyperaldosteronism and hypercortisolism has been increasingly attributed to the presence of multiple adrenocortical lesions. As shown in Supplementary Table S4, despite the scarcity of molecular confirmation in most reports, a few well-documented cases have provided molecular profiles. Fushimi et al. and Gao et al. reported cases of CPA with MAPM (12, 21). However, their clinical presentation was overt Cushing's syndrome with PA, which differs from our case. A similar finding was described by Teragawa et al., who reported a 58-year-old male with CPA and MAPM within a unilateral adrenal gland (22); however, molecular testing was not performed in that case. Among the three additional reported cases of APA/CPA that underwent molecular testing, all APAs harbored somatic *KCNJ5* mutations, and only one patient with overt Cushing's syndrome had a somatic *PRKACA* mutation in the CPA (23–25).

Notably, no previous study has reported a case similar to ours, which presents with concurrent somatic *GNAS* and *CACNA1D* mutations. Mechanistically, activating *GNAS* mutations drive cortisol overproduction and cell proliferation via constitutive activation of the cAMP/PKA signaling pathway, explaining the cortisol-dominant pathological phenotype of the tumor (26, 27). Meanwhile, the *CACNA1D* mutation lowers the activation threshold of voltage-gated calcium channels, leading to sustained calcium influx and subsequent upregulation of CYP11B2 expression. This likely underlies the clinical manifestations of PA and the observed MAPM/APN (28, 29). MAPM/APN can be found in the normal adrenal cortex and in the adrenal cortex near APA (30, 31). An emerging concept suggests that primary aldosteronism can originate from MAPM rather than conventional adrenal cortical adenomas (32). Although some studies have speculated that MAPM could represent a prodromal lesion of APA, the molecular changes in the two are often different (4, 33). The most common molecular change in APA is the *KCNJ5* mutation, whereas in MAPM, it is the *CACNA1D* mutation (34), indicating that the relationship and underlying mechanisms require further investigation. In our case, the patient's serum aldosterone level remained high 2 months after right adrenalectomy, indicating that a similar microscopic MAPM/APN may also exist in the left adrenal gland. This finding is consistent with the results of AVS, which indicated that the left adrenal gland was the dominant side for aldosterone secretion. We hypothesized that a similar microscopic MAPM/APN might also exist in the left adrenal gland to account for this persistent aldosterone excess. However, without histological confirmation from the contralateral gland, the presence of left-sided MAPM/APN remains uncertain. Therefore, based on the resected tissue, we can confirm that the multiple aldosterone-producing adrenocortical micronodules in the right adrenal gland contributed to the elevated serum aldosterone levels; however, the exact pathological substrate driving the persistent contralateral secretion remains unknown.

 To the best of our knowledge, the *CACNA1D* p.F1131L (c.3393C > G) variant has not been previously reported in cases of primary aldosteronism or aldosterone-producing adrenal lesions. However, somatic mutations in *CACNA1D* are widely recognized as the primary genetic cause of APCCs (also referred to as MAPM) (35). Structurally, the p.F1131L variant is located in exon 27, which encodes the highly conserved S6 segment of Domain IV of the calcium channel. This specific region plays a pivotal role in channel gating and function. Notably, other pathogenic mutations situated within this exact structural domain, such as p.F1147L, have been well-documented in the literature (4). Given their shared localization within the critical Domain IV S6 segment, it is highly plausible that the novel p.F1131L variant exerts functional consequences similar to those of p.F1147L, specifically lowering the activation threshold of voltage-gated calcium channels and leading to autonomous aldosterone production. Furthermore, our patient exhibited definitive histological evidence of APCCs/MAPM, providing a strong genotype–phenotype correlation that further supports the presumed pathogenicity of this variant. A review of population databases also revealed that this variant had an extremely low or absent allele frequency, and multiple *in silico* prediction algorithms (e.g., PolyPhen-2, Sorting Intolerant From Tolerant (SIFT)) predicted that this missense change is deleterious. Nevertheless, we acknowledge a critical limitation of our study: direct *in vitro* functional evidence (such as electrophysiological validation) confirming a gain-of-function effect of this specific p.F1131L substitution is currently lacking. Consequently, we cautiously refer to this alteration as a potential pathogenic variant, rather than a definitively established driver mutation.

The identification of the *GNAS* p.R201H mutation in our case is highly consistent with the patient's clinical presentation of MACS (16). The *GNAS* gene encodes the stimulatory G-protein alpha subunit (Gs*α*), and the p.R201H variant is a well-established activating hotspot mutation. As documented in the existing literature, this specific missense mutation inhibits the intrinsic GTPase activity of Gs*α*, leading to constitutive activation of the cAMP/PKA signaling pathway, which is a fundamental driver of adrenocortical cell proliferation and autonomous cortisol overproduction (26). Notably, recent genomic characterization of CPAs has revealed a distinct genotype–phenotype correlation (27). Although somatic mutations in PRKACA are predominantly associated with overt Cushing's syndrome, activating GNAS mutations are significantly more frequent in adenomas associated with MACS. Therefore, the presence of the *GNAS* p.R201H mutation is not only biologically plausible in this setting but is also intricately linked to the pathogenesis of MACS, perfectly aligning with the cortisol-dominant pathological phenotype of the main macroscopic nodule observed in our patient.

Although our experimental findings from bulk sequencing confirmed the simultaneous presence of GNAS and CACNA1D mutations, the current data cannot definitively demonstrate a temporal sequence or clonal relationship between these alterations. As a theoretical hypothesis, we propose a “two-hit” or “stepwise progression” model for the development of this lesion. Initially, a somatic mutation in *GNAS* may have triggered the activation of the cAMP/PKA signaling pathway, promoting the proliferation of adrenocortical cells and the formation of a macroscopic nodule primarily dedicated to cortisol production (CYP11B1+). Subsequently, a “second hit” in the form of a *CACNA1D* mutation, a gene widely recognized as a putative driver of aldosterone autonomy, might have occurred within this proliferative background and in adjacent cell clusters. Alternatively, it is highly possible that the two mutations occurred in completely different adrenal cell populations, representing spatially distinct molecular events. Although our single-block bulk tissue analysis precludes definitively assigning each variant to a specific pathological component, we hypothesize that the *GNAS* mutation likely drives the expansion of the large cortisol-producing nodule, whereas the *CACNA1D* mutation may represent a spatially distinct molecular event residing within the CYP11B2-positive MAPM included in the sampled tissue. This case highlights the value of multi-gene molecular testing in adrenocortical tumors with mixed hormonal features, which not only helps clarify the pathogenesis but also provides new insights into the clonal evolution and heterogeneity of adrenocortical lesions.

We acknowledge several limitations in our study. First, as a single-case report, the broader generalizability of these dual molecular findings in concurrent PA and MACS requires validation in larger cohorts. Second, while structurally and computationally supported, we lacked direct *in vitro* functional validation (e.g., electrophysiological studies) to definitively confirm the gain-of-function effect of the novel *CACNA1D* p.F1131L variant. Finally, the absence of lesion-specific molecular characterization through microdissection restricts our ability to unequivocally map *GNAS* and *CACNA1D* mutations to their exact cellular origins.

## 4 Conclusion

Here, we reviewed the literature on adrenal cortical adenomas in patients with PA and MACS. We also report a rare case of somatic mutations in both *GNAS* and *CACNA1D* within a unilateral adrenal lesion encompassing CPA and MAPM/APN. Although the lack of lesion-specific molecular characterization through microdissection represents a limitation of our study, we hypothesize that these variants are potential key drivers of concurrent PA and MACS. The atypical clinical and laboratory manifestations of combined PA and MACS can complicate the diagnosis. Based on previous reports and the findings of our case, independent hormone secretion by two distinct adrenal cortical lesions may serve as the primary explanation for the co-secretion of cortisol and aldosterone, whereas there is a lack of reliable molecular evidence supporting co-secretion from a single tumor. Pathologists should not only focus on the nodule itself when sampling lesions in one adrenal gland but also on pathological changes in the surrounding adrenal tissue, especially when clinical findings suggest abnormalities in both types of hormones. Moreover, our findings align with the emerging view that primary aldosteronism can originate from MAPM and not only from conventional APA.

## Data Availability

The original contributions presented in this study are included in the article/Supplementary Material. Further inquiries can be directed to the corresponding author.

## Supplementary material

Supplementary Figure S1 Timeline summarizing the patient's major clinical events. The chronological sequence outlines the comprehensive endocrine diagnostic work-up following admission, which included a simple standing test, captopril challenge test, AVS, and a dexamethasone suppression test. This diagnostic phase culminated in laparoscopic right adrenal resection. The final milestone highlights the 11-month postoperative follow-up, demonstrating well-controlled blood pressure (BP) maintained with Nifedipine 20 mg and Amlodipine 5 mg daily.

Supplementary Figure S2 Somatic missense mutations were detected in the *GNAS* and *CACNA1D* genes in the patient's right adrenal gland. **(A)** *GNAS* (exon8 c.602G > A, p.R201H). **(B)** *CACNA1D* (exon27 c.3393C > G, p.F1131L).
