# Supplementary material for: Case Report: Somatic GNAS and CACNA1D mutations responsible for adrenal cortisol-producing adenoma with multiple aldosterone-producing micronodules—concurrence of primary aldosteronism and mild autonomous cortisol secretion
Source: Front Med (Lausanne). 2026 Sep 24;13:1949358. doi: 10.3389/fmed.2026.1949358 (PMC13647305; doi:10.3389/fmed.2026.1949358)
Supplement: Supplementary file 1 [file Table1.docx]

Table S1 Results of adrenal venous sampling (AVS)

|  | Left adrenal vein 1 | Left adrenal vein 2 | Right adrenal vein 1 | Right adrenal vein 2 | Peripheral venous blood | Inferior vena cava |
| --- | --- | --- | --- | --- | --- | --- |
| Aldosterone (pg/mL) | 2753.44 | 1678.52 | 1205.88 | 1773.26 | 175.19 | 141.09 |
| Cortisal (μg/dL) | 27.66 | 18.25 | 302.6 | 307.16 | 8.11 | 7.49 |
| Aldosterone/Cortisal | 99.5 | 92.0 | 4.0 | 5.8 | 21.6 | 18.8 |
| MN (pg/mL) | 2085.8 | 1217.9 | 847.0 | 920.2 | 16.1 | 17.5 |
| Aldosterone/MN | 1.32 | 1.38 | 1.42 | 1.93 | 10.88 | 8.06 |
| Cortisal/MN | 0.013 | 0.015 | 0.36 | 0.33 | 0.5 | 0.43 |

MN, normetanephrine.
