# Supplementary material for: Case Report: Somatic GNAS and CACNA1D mutations responsible for adrenal cortisol-producing adenoma with multiple aldosterone-producing micronodules—concurrence of primary aldosteronism and mild autonomous cortisol secretion
Source: Front Med (Lausanne). 2026 Sep 24;13:1949358. doi: 10.3389/fmed.2026.1949358 (PMC13647305; doi:10.3389/fmed.2026.1949358)
Supplement: Supplementary file 2 [file Table2.docx]

Table S2 Antibodies for immunohistochemical staining

| Antibody | Clone | Producer | Antibody dilution |
| --- | --- | --- | --- |
| Inhibin | R1 | Agilent (Singapore) | Ready-to-use |
| MelanA | A103 | Agilent (Singapore) | Ready-to-use |
| P53 | D0-7 | Agilent (Singapore) | Ready-to-use |
| SF-1 | EP434 | Zhongshan Golden Bridge (Beijing, China) | Ready-to-use |
| CD56 | 123C3 | Agilent (Singapore) | Ready-to-use |
| CYP11B1 | ab197908 | Abcam (Cambridge, UK) | 1:3000 |
| CYP11B2 | ab168388 | Abcam (Cambridge, UK) | 1:3000 |
| CyclinD1 | EP12 | Agilent (Singapore) | Ready-to-use |
| STAR | ab203193 | Abcam (Cambridge, UK) | 1:200 |
| Ki-67 | MIB-1 | Agilent (Singapore) | Ready-to-use |
