# Supplementary material for: Case Report: Somatic GNAS and CACNA1D mutations responsible for adrenal cortisol-producing adenoma with multiple aldosterone-producing micronodules—concurrence of primary aldosteronism and mild autonomous cortisol secretion
Source: Front Med (Lausanne). 2026 Sep 24;13:1949358. doi: 10.3389/fmed.2026.1949358 (PMC13647305; doi:10.3389/fmed.2026.1949358)
Supplement: Supplementary file 3 [file Table3.docx]

Table S3 List of 45 genes included in the genetic panel

| Genes | Genes | Genes | Genes |
| --- | --- | --- | --- |
| AIP | TSC2 | SDHC | PRKAR1A |
| CACNA1D | ARID1A | VHL | SETD2 |
| EIF1AX | CDKN1B | ATM | ATP2B3 |
| KRAS | EZH2 | CTNNB1 | DNMT3A |
| RET | MEN1 | HRAS | KMT2A |
| TP53 | SDHB | PRKACA | PTCH1 |
| ALK | USP8 | SDHD | SF3B1 |
| CDC73 | ARMC5 | YY1 | ATRX |
| EPAS1 | CSDE1 | ATP1A1 | EGLN1 |
| MAX | GNAS | DAXX | KMT2D |
| SDHA | NF1 | KCNJ5 | RB1 |
| TMEM127 |  |  |  |
