# Supplementary material for: Case Report: Somatic GNAS and CACNA1D mutations responsible for adrenal cortisol-producing adenoma with multiple aldosterone-producing micronodules—concurrence of primary aldosteronism and mild autonomous cortisol secretion
Source: Front Med (Lausanne). 2026 Sep 24;13:1949358. doi: 10.3389/fmed.2026.1949358 (PMC13647305; doi:10.3389/fmed.2026.1949358)
Supplement: Supplementary file 4 [file Table4.docx]

**Table S4** Case Reports of unilateral or bilateral aldosterone-producing adenoma combined with cortisol-producing adenoma (A/CPA)

| Gender | Age | Hypertension | Location | Clinical diagnosis | Pathological diagnosis | Molecular findings | Ref |
| --- | --- | --- | --- | --- | --- | --- | --- |
| M | 30 Y | Yes | Bilateral | CS and PA | Left CPA, right APA | / | ^11^ |
| FM | 67 Y | Yes | Right | Overt CS and PA | CPA and APCCs | / | ^21^ |
| FM | 36 Y | Yes | Bilateral | CS and PA | Left APA, right CPA | Left *KCNJ5* somatic mutation | ^23^ |
| FM | 45 Y | No | Bilateral | Overt CS and PA | Right Co-secretory tumors, left CPA and APN, bilateral APCCs | / | ^12^ |
| FM | 62 Y | Yes | Right | Subclinical CS and PA | Three tumours, one was APA, the others were CPA | / | ^9^ |
| M | 54 Y | Yes | Bilateral | Subclinical CS and PA | Left CPA, right APA | Right *KCNJ5* somatic mutation | ^24^ |
| M | 56 Y | Yes | Bilateral | Subclinical CS and PA | Bilateral AIMAH, left APA | / | ^35^ |
| M | 52 Y | Yes | Bilateral | Subclinical CS and PA | Bilateral AIMAH, left APA | / | ^36^ |
| FM | 49 Y | Yes | Bilateral | Subclinical CS and PA | / | / | ^14^ |
| FM | 31 Y | Yes | Bilateral | Subclinical CS and PA | Left CPA, right APA | / | ^37^ |
| FM | 50 Y | Yes | Bilateral | CS and PA | Left APA, no right adrenalectomy | / | ^38^ |
| FM | 49 Y | Yes | Right | Overt CS and PA | Two tumours, one was APA, the another was CPA | *KCNJ5* and *PRKACA* somatic mutations | ^25^ |
| M | 58 Y | Yes | Right | Subclinical CS and PA | CPA with APCCs in the adjacent non-neoplastic adrenal | */* | ^22^ |

M, male; FM, female; Y, years of age; CS, Cushing’s syndrome; PA, primary aldosteronism; CPA, cortisol-producing adenoma; APA, aldosterone-producing adenoma; APCCs, aldosterone-producing cell clusters; AIMAH, ACTH-independent adrenocortical macronodular hyperplasia; APN, aldosterone-producing nodule

# Reference

11. Ren K., Wei J., Liu Q., et al. Hypercortisolism and primary aldosteronism caused by bilateral adrenocortical adenomas: a case report. *BMC Endocr Disord.* 2019; *19* (1), 63.

21.	Fushimi Y., Tatsumi F., Sanada J., et al. Concurrence of overt Cushing's syndrome and primary aldosteronism accompanied by aldosterone-producing cell cluster in adjacent adrenal cortex: case report. *BMC Endocr Disord.* 2021; *21* (1), 163.

23.	Zhao L., Wan J., Wang Y., et al. Different cell compositions and a novel somatic KCNJ5 variant found in a patient with bilateral adrenocortical adenomas secreting aldosterone and cortisol. *Front Endocrinol (Lausanne).* 2023; *14*, 1068335.

12.	Gao H., Li L., Chen F., et al. Bilateral co-secretory lesions presenting with coexisting Cushing syndrome and primary aldosteronism: a case report. *BMC Endocr Disord.* 2023; *23* (1), 263.

9.	Okura T., Miyoshi K., Watanabe S., et al. Coexistence of three distinct adrenal tumors in the same adrenal gland in a patient with primary aldosteronism and preclinical Cushing's syndrome. *Clin Exp Nephrol.* 2006; *10* (2), 127-30.

24.	Zhang Y., Tan J., Yang Q., et al. Primary aldosteronism concurrent with subclinical Cushing's syndrome: a case report and review of the literature. *J Med Case Rep.* 2020; *14* (1), 32.

35.	Tokumoto M., Onoda N., Tauchi Y., et al. A case of Adrenocoricotrophic hormone -independent bilateral adrenocortical macronodular hyperplasia concomitant with primary aldosteronism. *BMC Surg.* 2017; *17* (1), 97.

36.	Hayakawa E., Yoshimoto T., Hiraishi K., et al. A rare case of ACTH-independent macronodular adrenal hyperplasia associated with aldosterone-producing adenoma. *Intern Med.* 2011; *50* (3), 227-32.

14.	Hu L., Ji W., Guo M., et al. Case Report: Primary Aldosteronism and Subclinical Cushing Syndrome in a 49-Year-Old Woman With Hypertension Plus Hypokalaemia. *Front Cardiovasc Med.* 2022; *9*, 911333.

37.	Lee S. E., Kim J. H., Lee Y. B., et al. Bilateral Adrenocortical Masses Producing Aldosterone and Cortisol Independently. *Endocrinol Metab (Seoul).* 2015; *30* (4), 607-13.

38.	Han J. E., Yoo S., Lee S. A., et al. Bilateral adrenal adenomas with autonomous cortisol secretion from both glands and autonomous aldosterone secretion from the left adrenal: a case report. *J Yeungnam Med Sci.* 2025; *42*, 33.

25.	Nanba K., Omata K., Tomlins S. A., et al. Double adrenocortical adenomas harboring independent KCNJ5 and PRKACA somatic mutations. *Eur J Endocrinol.* 2016; *175* (2), K1-6.

22.	Teragawa H., Oshita C., Orita Y., et al. Primary aldosteronism due to bilateral micronodular hyperplasia and concomitant subclinical Cushing's syndrome: A case report. *World J Clin Cases.* 2021; *9* (5), 1119-1126.
